# A feedback control principle common to several biological and engineered systems

**DOI:** 10.1098/rsif.2021.0711

**Published:** 2022-03-02

**Authors:** Jonathan Y. Suen, Saket Navlakha

**Affiliations:** Cold Spring Harbor Laboratory, Simons Center for Quantitative Biology, Cold Spring Harbor, NY, USA

**Keywords:** feedback control, biological distributed algorithms, ant colonies, neural circuits, cell size, ‌synaptic plasticity, networks

## Abstract

Feedback control is used by many distributed systems to optimize behaviour. Traditional feedback control algorithms spend significant resources to constantly sense and stabilize a continuous control variable of interest, such as vehicle speed for implementing cruise control, or body temperature for maintaining homeostasis. By contrast, discrete-event feedback (e.g. a server acknowledging when data are successfully transmitted, or a brief antennal interaction when an ant returns to the nest after successful foraging) can reduce costs associated with monitoring a continuous variable; however, optimizing behaviour in this setting requires alternative strategies. Here, we studied parallels between discrete-event feedback control strategies in biological and engineered systems. We found that two common engineering rules—additive-increase, upon positive feedback, and multiplicative-decrease, upon negative feedback, and multiplicative-increase multiplicative-decrease—are used by diverse biological systems, including for regulating foraging by harvester ant colonies, for maintaining cell-size homeostasis, and for synaptic learning and adaptation in neural circuits. These rules support several goals of these systems, including optimizing efficiency (i.e. using all available resources); splitting resources fairly among cooperating agents, or conversely, acquiring resources quickly among competing agents; and minimizing the latency of responses, especially when conditions change. We hypothesize that theoretical frameworks from distributed computing may offer new ways to analyse adaptation behaviour of biology systems, and in return, biological strategies may inspire new algorithms for discrete-event feedback control in engineering.

## 1. Introduction

Homeostasis refers to the ability of a system to recover to a desired set point after being changed or perturbed [1]. In both biology and engineering, feedback control is used to adapt behaviour to changing conditions to achieve homeostasis or equilibrium. Traditionally, feedback control is applied in dynamical systems that provide a continuous variable as feedback [2], such as in cruise control to keep a vehicle at a constant speed, or in the homeostatic regulation of body temperature. However, some systems are regulated by feedback triggered by naturally discrete events. Examples of these events include an acknowledgement received when data are successfully transmitted over the Internet; or antennal interactions when a foraging ant returns to the nest with food. Critically, discrete-event feedback requires less communication and measurement resources compared to continuous feedback but also requires fundamentally different strategies to optimize behaviour.

The behaviour of discrete-event feedback systems is determined by how a control variable changes upon receipt of positive or negative feedback. The most common first-order response to feedback can be described as additive, in which a constant is added or subtracted to the variable, or multiplicative, where the variable is multiplied or divided by a constant. For example, on the Internet, if a server is sending data at some rate *r*, then upon positive feedback, the rate might increase to *r* + 1 (additive) or 2*r* (multiplicative). Similarly, upon negative feedback, the rate might decrease to *r* − 1 or *r*/2, respectively. In biology, an ant returning to the nest with food would trigger additional foragers to depart (since there is likely more food available), whereas an ant returning empty-handed would suppress the departure of additional foragers.

The challenge from a design standpoint is to understand how these seemingly simple differences in response rules affect the performance of the system. Performance can be measured in various ways, including how well available resources are used (e.g. it is inefficient to send data at a rate that is far below available bandwidth, or to have few foragers searching for food when plenty is available); how well resources are shared among cooperating agents or acquired by competing agents; and how quickly the system can respond to changes in resource availability. Importantly, the number of available resources (e.g. total bandwidth, total amount of food) is unknown to any individual agent. Although it may be intuitive to apply the same type of response to both types of feedback (e.g. add a small constant for positive feedback, subtract a small constant for negative feedback), it turns out that this is not always best.

Our aim in this review is to synthesize principles shared by biological and engineered systems to optimize behaviour in response to discrete-event feedback [3,4]. We first develop a basic model for distributed event-based feedback control in which a collection of agents need to share or acquire a resource. We then apply this framework to examples from three biological systems (foraging by harvester ants, cell size control and homeostasis, and rules for adaptive synaptic plasticity in the brain), as well as two engineered systems (bandwidth control on the Internet, and online decision-making in machine learning). From these systems, we show that two common rules—additive-increase multiplicative-decrease (AIMD), which adds a small constant to the control variable upon positive feedback, and multiplies the variable by a constant <1 upon negative feedback, and multiplicative-increase multiplicative-decrease (MIMD)—are used to achieve multiple goals, including efficient allocation of available resources, the fair or competitive splitting of those resources, minimization of response latency, and the ability to detect feedback failures. We end by discussing more sophisticated control rules that utilize prior history of the system or other complex system variables, and we highlight potential avenues for future cross-disciplinary collaboration.

## 2. A model of discrete-event feedback

We begin with a control variable of interest (*r*) whose value needs to be adjusted in response to discrete-event feedback. For example, a server on the Internet sends packets of data to a user at a rate *r*, and based on feedback from the user indicating whether the data were received or dropped in transit, the transmission rate *r* is either increased or decreased. For a harvester ant colony, *r* could indicate how many foragers leave the nest in search of food, and this rate is regulated based on the number of successful foragers returning to the nest with food. We assume feedback received is only 1-bit: 0 (negative) or 1 (positive).

There are two global constraints that restrict the value that *r* should take. First, *capacity* is the total amount *C* of resource that is available. On the Internet, the resource is bandwidth, which limits the maximum transmission rate; for ants, the resource is the rate at which food becomes available in the environment, which limits the number of ants that ought to be foraging. Attempting to consume more resources than are available leads to inefficiency: if data are sent at an excessive rate, then data packets will be dropped in transit, which wastes bandwidth; if more ants forage than food available, then ants unnecessarily waste energy. Conversely, idle data links and uncollected food also represent a wasted resource. A system where the total rate matches capacity—i.e. $\sum_{i}r_{i}=C$, over all users *i*—is said to be optimally *efficient*. Moreover, as $\sum_{i}r_{i}$ approaches or exceeds *C*, the time to accomplish a task may increase; data packets start waiting in queues, and ants start spending more time searching for food. Thus, for many systems, an increase in *latency*, which is the time delay between the start of a task and receiving feedback, acts as an early warning against overload.

The second global constraint is that *n* users either cooperate or compete for the resources. On the Internet, there are millions of users sending and receiving data simultaneously, and all data transmissions go through shared networking links. For harvester ants, there are multiple colonies living in the habitat that need to acquire food. In a cooperative system, agents (e.g. Internet users) desire to share resources equally, which is achieved when all the rates *r*_*i*_ are equal (called *fairness*). By contrast, in a competitive system, agents (e.g. ant colonies) seek the largest share possible.

Critically, the values of *C* and *n* are unknown to any individual agent, and may vary unpredictably with time. Feedback implicitly encodes the relationship between an agent’s individual rate and the two global variables. Intuitively, if agent *i* receives positive feedback, then the current value of *r*_*i*_ should be increased, and vice versa for negative feedback. But how much should the variable be increased or decreased?

Four common strategies for how the *r*_*i*_ variables can be modified in response to feedback are:

1. *Additive*. A constant is added to or subtracted from the value of the current variable.
2. *Multiplicative*. The variable is multiplied or divided by a constant.
3. *Functional*. The variable is modified by some more complex function of its current value (e.g. a quadratic or cubic function).
4. *Time-dependent*. The new value of the variable depends on both the current value and a history of recent changes to the variable.

Here, we focus on the first two strategies (additive and multiplicative), which are simple, require no memory, and are found in a broad range of biological and engineered systems, as we will see. The latter two strategies (functional and time-dependent) generalize the first two, and due to their increased complexity, are often found in empirically designed systems

Formally, given the current value of an agent’s variable $r_{i}^{t}$ at time *t*, upon receipt of feedback, its value is updated to:

$$r_{i}^{t+1}=\left\{ \begin{array}{cc} r_{i}^{t}+I_{a},I_{a}>0\; & \text{additive increase (AI) }\;\\ r_{i}^{t}\times I_{m},I_{m}>1\; & \text{multiplicative increase (MI) }\;\\ r_{i}^{t}-D_{a},D_{a}>0\; & \text{additive decrease (AD) }\;\\ r_{i}^{t}\times D_{m},0<D_{m}<1\; & \text{multiplicative decrease (MD).}\; \end{array}\right.$$

*I*_*a*_ and *I*_*m*_ are additive and multiplicative increase constants, and *D*_*a*_ and *D*_*m*_ are additive and multiplicative decrease factors, respectively. An increase rule is applied in response to positive feedback and a decrease rule is applied to negative feedback.

### 2.1. Properties of additive and multiplicative rules

There are four possible combinations of rules: additive-increase additive-decrease (AIAD), additive-increase multiplicative-decrease (AIMD), multiplicative-increase additive-decrease (MIAD) and multiplicative-increase multiplicative-decrease (MIMD). One striking result in engineering and communication network theory is that, in cooperative systems, efficiency and fairness can only be achieved with AIMD. This result requires detailed mathematical proof [5]; however, later in the paper, we provide intuition of why this is true using a simple case with two users (figure 5). For competitive systems, MIMD can also achieve efficiency, but instead of achieving fairness, MIMD preserves the relative ratios of the *r*_*i*_’s [6,7], or can be used to favour better-performing agents at the expense of those with worse performance. The two other variants have less favourable properties: AIAD can reach efficiency without preserving fairness, and MIAD is not efficient and is maximally unfair because it provides all the resources to the agent with the initially higher allocation [8]. We are not aware of any examples of multi-user AIAD and MIAD systems in either biology or engineering.

Later, we will provide intuition for why these rules have the properties that they do, but we first provide several examples from biology and engineering that instantiate this discrete-event feedback control framework and how the rules above are used to optimize behaviour.

## 3. Examples of discrete-event feedback in biology

We next look at three biological examples of discrete-event feedback control (table 1). These examples range from the organism level (harvester ant foraging) to the cellular level (cell size control) to the molecular level (synaptic plasticity in the brain), and they encompass both cooperative and competitive scenarios.

**Table 1.** Summary of discrete-event feedback control rules used by biological and engineering systems.

system	algorithm	efficient?	fair?	compete?	latency?	timeout?	slow start?
*biological*							
harvester ant foraging	MIMD	✓	✕	✓	✓	✓	✓
cell-size homeostasis	AIMD	✓	✓	✕	✕	?	✓
brain: novelty detection	AIMD	✓	✓	✕	✕	✕	✕
brain: homeostatic plasticity	MIMD	✓	✕	✓	✕	✕	✕
brain: STDP	Mixed	✓	✓	✓	✓	✕	✕
*engineered*							
Internet flow control/TCP	AIMD	✓	✓	✕	✓	✓	✓
machine learning weights update	MIMD	✕	✕	✓	✕	✕	✕

In the examples below, we highlight experimental results that describe how different rules are applied. Our contribution is not the discovery of these rules but rather casting them under a single framework and relating them to global system properties. In addition, we highlight the core features of these systems, and their relationship to these rules. Later, when we synthesize common features across these systems, we discuss additional complexities where some deviation from these rules are observed in specific circumstances (e.g. during initialization or restart).

### 3.1. Foraging behaviour by harvester ants (figure 1)

Red harvester ants (*Pogonomyrmex barbatus*) inhabit desert environments and obtain the food and water necessary for survival by foraging for seeds [9]. Seeds are scattered in the environment by wind and rain, and multiple colonies compete for these limited resources [10,11]. Ants experience desiccation while foraging in the heat outside their nest, and the water lost is only replenished by metabolizing fats from seeds they harvest [12]. Thus, to maximize the net gain in resources, the rate of ants foraging needs to match the time-varying availability of seeds.

**Figure 1.**
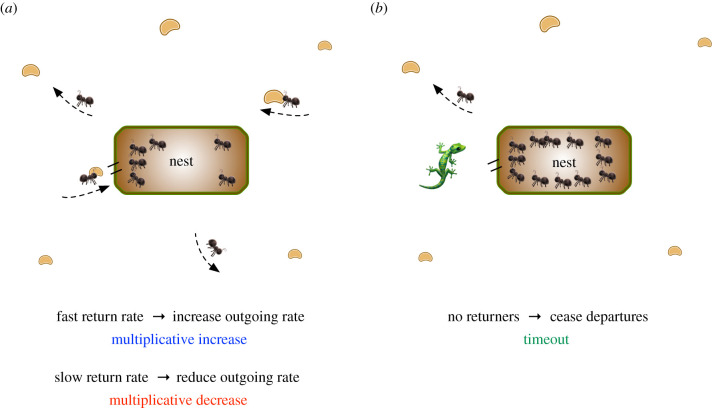
Distributed foraging rates in harvester ants. (*a*) Foraging ants search for food sources (seeds) in the environment. When a successful forager returns to the nest with a seed, it provides feedback via antennae contact with ants queued at the entrance of the nest. These ants then leave the nest to forage themselves. The sooner foragers return with seeds, the faster outgoing ants leave the nest, and vice versa, in a multiplicative manner. (*b*) Example of timeout. If foragers do not return to the nest for a long enough time (in this example, 20 min), then a timeout occurs, and no further ants leave the nest. This could be caused, for example, by a predator in the environment.

In this example, there are *n* colonies in the environment that compete for resources. Ants from colony *i* deploy from the nest to forage at a rate *r*_*i*_. *C* corresponds to the rate at which seeds become available in the environment, which can vary unpredictably.

To maximize fitness of the species (i.e. a collection of colonies), one goal for the system is to achieve efficiency; i.e. the rate of ants leaving all nests should equal *C*. If foraging rates exceed the rate at which food becomes available, then many ants would return ‘empty-handed’ [13,14], resulting in little or no net gain in colony resources. If foraging rates are lower than the food availability rate, then seeds would be left in the environment uncollected, meaning the seeds would either be lost to other colonies or be removed by wind and rain.

How do harvester ant colonies use feedback control to determine when, and how many, ants leave the nest in search for seeds? Feedback occurs at the entrance of the nest, where foraging ants carrying food return to the nest and interact with the queue of outgoing ants waiting to leave the nest (figure 1*a*). Experiments showed that returning foragers exchanged cuticular hydrocarbons via brief antenna interactions with ants waiting to depart the nest. When an ant forages, the composition of its cuticular hydrocarbons changes based on the time spent in the different temperature and humidity of the outside environment [12]. When ants waiting to depart were exposed to the odour of food and the modified cuticular hydrocarbons, their waiting time was shortened [15]. A similar effect was observed when the rate of forager return was experimentally manipulated [16,17]. Most harvester ants continue to search until they find food, only returning to the nest empty-handed after an hour, approximately three times longer than the average search time [16].

To avoid the time and resources wasted on an unsuccessful search, ants have evolved a system in which departure rates are adjusted based on sensing the time a returning ant spent outside the nest. This latency-based feedback is positive when foraging times are short, and negative when foraging times increase, allowing early detection of incipient food shortages.

Modelling of this process has found that the relationship between departing and arriving foraging rates in steady state is well captured as an MIMD system. Given the large number of distributed interactions, ants were modelled by Prabhakar *et al.* [18,19] as a distributed, stochastic system where the departure rates *r*_*i*_ are draws from a Poisson distribution,

$$r_{i}^{t}=\mathrm{Poisson}(\alpha_{i}^{t}),$$

where the time-dependent parameter

3.1 $$\alpha_{i}^{t}=\max(\alpha_{i}^{t-1}-dr_{i}^{t-1}+uA_{i}^{t}-\epsilon ,\underline{\alpha }).$$

Here, $\alpha_{i}^{t}$ represents the mean departure rate at time *t* for colony *i* and is determined by constants *d* > 0 and *u* > 0; $A_{i}^{t}$ is the number of incoming ants at time step *t*; *ε* is a decay term, which captures an observed lack of response when ants return very slowly to the nest, and is set to 0 in the model; and *α* acts as a small rate floor.

MIMD is manifested through the effect of the *u* and *d* constants. We show this by first simplifying the model by analysing the average rate ($r_{i}^{t}=\alpha_{i}^{t}$, removing the stochastic nature), and assuming the minimum rate floor (*α*) is never reached. To further avoid a stochastic model, we assume a foraging time, Δ*t*, which is fixed at any instant across all foraging ants, such that the arrival rate is equal to the departure rate Δ*t* time-steps in the past, i.e. $A_{i}^{t}=r_{i}^{t-\Delta t}$. This rate may nonetheless vary slowly as resource scarcity changes, and could be seen as a mean foraging time. We then can rewrite equation (3.1) to give us the departure rate as

$$\begin{array}{rl} r_{i}^{t} & =r_{i}^{t-1}-dr_{i}^{t-1}+ur_{i}^{t-\Delta t}\\ & =(1-d)r_{i}^{t-1}+ur_{i}^{t-\Delta t}. \end{array}$$

The *u* term increases the foraging rate by a multiplicative factor of the time-delayed rate. Thus, the growth in foraging rate depends on the time it takes for an ant to depart, find food and return to the nest (called round-trip time). The (1 − *d*) term acts as a constantly applied multiplicative decrease, which acts against the multiplicative increase. If ants return to the nest quickly, the increase process dominates, and when latency increases, the decrease term dominates. Thus, when the foraging time is short, the foraging rate grows rapidly, and conversely when foraging time is long, the foraging rate is slowed or ceased, in effect, acting as a *latency-sensitive* version of MIMD.

Thus, harvester ants evolved a simple discrete-event feedback control algorithm to adjust foraging rates based on food availability in uncertain environments. We later hypothesize why MIMD may have evolved in this competitive environment, as opposed to AIMD.

### 3.2. Cell size control and homeostasis (figure 2)

Organisms contain anywhere between a single cell (bacteria) to trillions of cells (plants and animals). Proper function and physiology of the organism depends on creating cells of the appropriate sizes [20,21]. Indeed, cell size affects numerous biological functions, including metabolism rates, molecular transport efficiency and mechanical properties of the cell [22,23]. Consequently, cell size affects the scales of subcellular compartments [24] and of tissues and organs [25].

**Figure 2.**
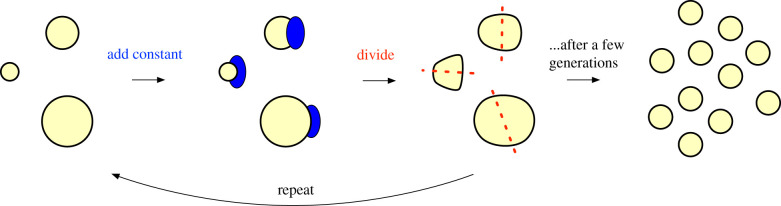
The ‘adder’ mechanism for cell size homeostasis. There are initially cells of various sizes. Each cell adds a constant volume to its existing size, and then divides in half. This process repeats for a few generations and exponentially converges to a state wherein all cells are of the same size.

The appropriate size of a cell also depends on its function. For example, red blood cells and sperm cells are very small because they navigate through tight spaces, whereas muscle cells are large because they need to generate and sustain high mechanical force. While variation in size across cell types is to be expected, the size of cells of the same type is typically more uniform [21], and abnormal variation in sizes within the same cell type has been linked to diseases, including cancer [20].

How do cells maintain size homeostasis? Since the molecular mechanisms controlling cell size in eukaryotic cells still remain largely elusive [21,26], here we focus on bacterial cell size, whose mechanisms are much better understood. In this example, there are initially *n* cells. Each cell *i* occupies space *r*_*i*_, and *C* corresponds to the total volume that the cells can occupy. Cell size depends on both growth and division processes, where growth increases cell size, and cell division divides the cell into two. These processes are inherently variable due to stochastic developmental events; furthermore, the lack of synchronization across cells means that the initial cell sizes over the population can be arbitrary.

Technological advances in live-cell imaging and single-cell tracking have revealed new insights into the mechanisms that cells use to converge to homeostasis and prevent size divergence [21]. In bacteria, the emerging model is called *adder* [27] (figure 2). In this model, a cell grows by adding a constant volume in each generation *t*; i.e. $r_{i}^{t}=r_{i}^{t-1}+I_{a}$, where *I*_*a*_ is a constant, irrespective of initial size. After growth, each cell divides by two; i.e. $r_{i}^{t}=r_{i^{\prime }}^{t}=r_{i}^{t-1}/2$, assuming symmetric division, with *i*′ representing the new cell born from the division. Under this model, cell size fluctuations decrease by 50% per generation; i.e. cell size homeostasis approaches *I*_*a*_ exponentially, starting from any initial state. For example, say there are two cells, $r_{i}^{0}=10$ and $r_{j}^{0}=1$ and let *I*_*a*_ = 4 and *D*_*m*_ = 1/2 (the latter variable corresponding to multiplicative decrease when cells divide by 2). Then, after four generations, the sizes become $r_{i}^{4}=4.38$ and $r_{j}^{4}=3.81$.

Mechanistically, the adder model requires two conditions, most commonly found in bacteria [27,28]: (a) cell division proteins are synthesized at the same rate as the growth of the cell and (b) once a threshold number of division proteins are synthesized, division begins. Thus, in this model, positive feedback (additive-increase, *I*_*a*_) is applied a constant number of times until the threshold is reached. When the threshold is reached negative feedback (multiplicative-decrease, *D*_*m*_) is applied and the cell is split into two.

From an engineering perspective, the adder model is similar to AIMD. In each generation, every cell adds a constant to its size, and then multiplicatively divides by two. While cells do compete for growth substrates, cells cannot outgrow their environment, otherwise, they will lose access to the nutrients needed for growth. Indeed, AIMD ensures that the total size of the population, after many generations, approaches the total volume available to occupy; i.e. that $\sum_{i}r_{i}=C$, providing efficiency. On the other hand, why might bacterial cells seek fairness (i.e. uniformity in the *r*_*i*_ values)? One idea is that bacteria must maintain a population of cells to survive. Populations enable numerous cooperative behaviours [29], such as quorum sensing, biofilm formation and shape formation [30]. Thus, while individual microbial cells do compete for resources, this must be balanced by the need to maintain a stable population. In addition, a cell’s growth rate can fluctuate, over cell cycles, based on the environment and nutrient availability; if not regulated, this can lead to instability and divergence in cell sizes over the population [31,32]. AIAD and MIAD do not converge to fairness, and in fact, MIAD would be maximally unfair, meaning that one cell would grow to take over all the resources [8]. Furthermore, MIMD would preserve this instability because it preserves the relative ratios of the *r*_*i*_ values. On the other hand, AIMD would dampen the effects of fluctuations, leading to a stable population.

Recently, mixed strategies to control cell size have also been discovered. For example, plant cells (i.e. shoot apical meristem in *Arabidopsis*) use a hybrid model where cell size increases multiplicatively for the first 80% of the cell cycle, and then additively for the final 20% [33]. In instances where $r_{i}^{0}$ is initialized to a size that is far from its homeostatic state, the initial multiplicative increase results in faster convergence; this is related to a property called slow-start, which we will describe later. Indeed, if asymmetric division occurs, then the smaller sister grows at a faster rate than the larger sister [33].

There are caveats to the rules discussed above, especially with regards to how growth and division vary with environmental conditions, and with regards to mammalian cells, which use more sophisticated and non-local sensing mechanisms to monitor growth. For example, the sizer model, where cells grow to a fixed absolute size before dividing, is found in some cells of eukaryotic species (e.g. yeast [34]) and requires elaborate mechanisms for cells to sense their own size and determine when division begins, and these mechanisms remain largely unknown. Conversely, the timer model, where a cell grows for a fixed period of time before dividing, does not compensate for growth rate variability at all, and requires a mechanism to measure time, and thus it has become less popular. Nonetheless, experiments in bacterial systems have demonstrated the use of simple feedback control algorithms to maintain cell size homeostasis.

### 3.3. Synaptic plasticity in the brain

Synapses are core structures in the brain that mediate communication signalling between neurons. The strength of synapses can be modulated by learning mechanisms (called plasticity), and thus synapses form one basis of computation and memory in the brain. The most common model of their action involves one or more pre-synaptic neurons that transmit impulses through synapses, which can then combine to trigger the firing of an impulse from a post-synaptic neuron.

Next, we discuss three examples of synaptic plasticity rules and their relationships to feedback control algorithms. These examples are novelty detection, homeostatic plasticity and spike-timing-dependent plasticity (STDP), which highlight different goals, including minimization of latency, fairness and efficient scaling.

#### 3.3.1. Novelty detection via reinforcement feedback (figure 3*a*)

A recent example of AIMD in the brain comes from synaptic-level physiology in the fruit fly olfactory system [35–37]. When an odour is presented to a fly, a small set of neurons, called Kenyon cells (KCs), fire in response. There are about 2000 KCs in total, but only about 5% (100 cells) fire for any odour [38–40]. This sparsity generates non-overlapping representations for different odours, which makes it easier to discriminate odours [41] and associate them with different behaviours [42].

**Figure 3.**
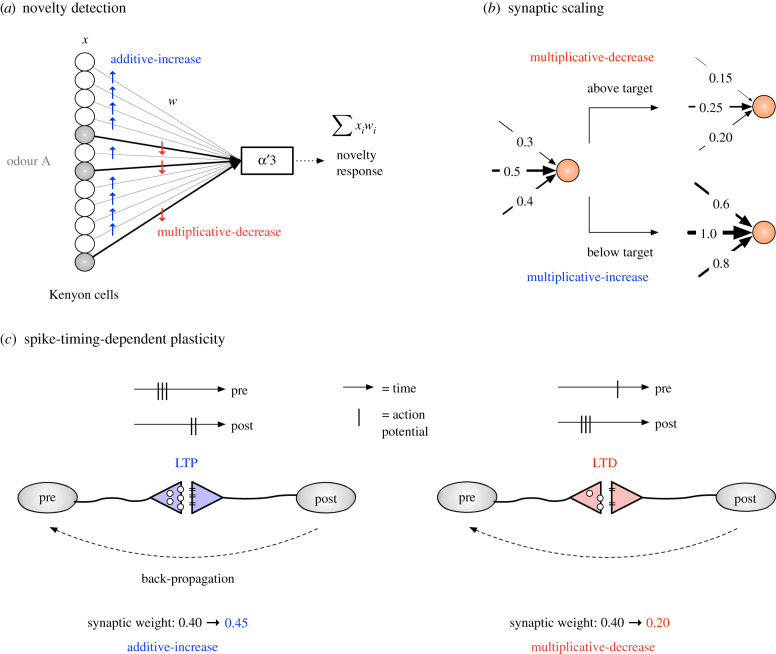
Synaptic plasticity in the brain. (*a*) Weights of synapses active for the odour are multiplicatively decreased, to quickly reduce the novelty of the odour. Weights of synapses inactive for the odour are additively increased, since the corresponding odours encoded by these synapses are now slightly more novel than before. (*b*) When neurons persistently fire significantly above or below the target firing rate, homeostatic plasticity mechanisms kick in. Neurons firing above the target rate induce a multiplicative decrease in synapse sizes, and a multiplicative decrease for neurons firing below the target rate. (*c*) Left: long-term potentiation (LTP) at a synapse occurs when the pre-synaptic neuron fires which leads to the post-synaptic firing soon after. The horizontal bar indicates time, and the vertical bars indicate the times when the pre- or post-synaptic neuron fires. LTP is additive, meaning that the synaptic weight increases by small constant (in the example, 0.05). The feedback mediating this change occurs, for example, due to a back-propagating action potential, which causes an increase in the pre-synaptic release probability or number of post-synaptic receptors. Right: long-term depression (LTD) at a synapse occurs when the pre-synaptic cell fires after the post-synaptic cell. In this case, the synaptic weight decreases multiplicatively (in the example, dividing by two).

Hattori *et al.* [43] recently showed how fruit flies use these sparse representations for determining the novelty of an odour. Novel odours are those which have not been experienced before by the organism, or that have been experienced a very long time ago. The latter implies some natural memory decay or ‘forgetting’ experienced by the organism. Determining whether an odour is novel, or has not been experienced in a long time, is an important neural computation that alerts organisms to new and potentially salient events [44]. An odour’s novelty increases slowly over time when not observed, and decreases sharply when observed. Hattori *et al.* [43] found that a single output neuron (called MBON *α*′3) calculates the novelty of an odour based on input received from KCs.

In this example, there are *n* KCs {*x*_1_, *x*_2_, …, *x*_*n*_}, and {*r*_1_, *r*_2_, …, *r*_2000_} correspond to the synaptic weights between the KCs and the MBON. These synapses do not compete with each other, but they do share common resources *C* (e.g. a pool of neurotransmitters [45], a number of receptor proteins), which are required to implement weight changes. These resources are not as strictly fixed as in prior examples, but they are not unbounded either.

The MBON’s novelty calculation can be computed as [46]: $\sum_{i}x_{i}r_{i}$. For any given odour, only 100 of the *x*_*i*_ are active (non-zero), and the rest are zero. Initially, all *r*_*i*_ are 1. When an odour arrives, some KCs fire in response, and those KCs strongly drive the activity of the MBON. Hence, the odour is novel. After the odour arrives, there is feedback from a dopamine neuron that modifies the weights of all the KC→MBON synapses. Synapses from KCs that were inactive for the odour undergo an increase in their weight, and synapses from active KCs undergo a weight decrease.

In a re-analysis of data by Hattori *et al.* [43], Dasgupta *et al.* [46] modelled this as

$$r_{i}^{t+1}=\left\{ \begin{array}{cc} r_{i}^{t}+I_{a}\; & \text{if the }i\mathrm{th}\text{ KC is not active for the odour}.\;\\ r_{i}^{t}\times D_{m}\; & \text{if the }i\mathrm{th}\text{ KC is active for the odour}.\; \end{array}\right.$$

The first rule additively increases the weights of synapses inactive for the odour; this effectively increases the novelty for all odours that were not observed, which are encoded by inactive KCs. Intuitively, one time-step has passed since those odours were observed, which means they are slightly more novel at time *t* + 1 than they were at *t*. The second rule multiplicatively decreases the novelty of the observed odour, so that if it is immediately experienced again, it would be much more familiar.

Why might AIMD be desirable for calculating novelty? First, AIMD converges to fairness, which means that all *r*_*i*_ are approximately equal (i.e. all odours have equal novelty), under a random sequence of odours. Over time, this property would be maintained regardless of initial conditions. Second, AIMD ensures efficiency, which may be desirable since synaptic changes require using shared, limited resources.

These rules also coincide with two intuitive properties of novelty from a biological standpoint. First, recovery from familiarity back to novelty should be relatively slower (additive). Otherwise, very soon after observing an odour, the odour would become novel again, limiting the timescale over which novelty can be integrated. Second, decay after initial exposure to an odour should be aggressive (multiplicative). If decay were slow, then the novelty signal would persist over several successive presentations of an odour, which may unnecessarily burden attention.

This example relates to the broader literature of homeostasis [1] and perfection adaptation [47,48], where cells deviate from some baseline (e.g. due to a stimulus or perturbation) and then use integral feedback to re-establish the baseline responsiveness.

#### 3.3.2. Mechanisms for homeostatic plasticity (figure 3*b*)

Homeostasis refers to the ability of a system to recover to some set point after being perturbed [1], which is analogous to the concept of stability in engineering. In the brain, homeostatic plasticity mechanisms [49] are used to stabilize network activity to remain in preferred ranges [50]. For example, if neuron *u* is connected to neuron *v* and drives it to fire, the synapse between them may get strengthened (e.g. via long-term potentiation, LTP). Then, the next time *u* fires, it is even more likely that *v* fires, and this positive feedback loop can lead to run-away activity. Similarly, if the synapse weakens (e.g. via long-term depression, LTD), then *v* is less likely to fire next time, and this negative feedback can lead to insufficient activity. The job of homeostasis is to prevent neurons from being both over-used (hyperactive) and under-used (hypoactive) [51]. Disruption of homeostasis mechanisms can lead to neurological disorders [52–57], indicating their importance for normal brain function.

In this example, there are *n* synapses on the dendrites (inputs) of a neuron, and *r*_*i*_ corresponds to the synaptic weight of the *i*th synapse. *C* corresponds to the total weight that can be allocated among the *n* synapses. As above, individual weights are determined competitively, by acquiring resources, such as neurotransmitters, or receptor proteins; moreover, it is well known that many neurons preserve an approximate lognormal distribution of synaptic weights over long time scales, regardless of their activity level [58,59]. Thus, the sum of the *r*_*i*_ is approximately constant.

Experimental analysis of homeostatic plasticity mechanisms has uncovered a rule, called *synaptic scaling* [51]. The idea is as follows: say, each neuron has a target firing rate at which it prefers to fire, and that the synapses of the neuron undergo learning-related changes (e.g. LTP, LTD) that shift the actual firing rate away from the target. If the neuron starts firing above its target rate, then all of its incoming (excitatory) synapses are downscaled (i.e.multiplied by some factor, 0 < *D*_*m*_ < 1). On the other hand, if a neuron fires below its target, then all its excitatory synapses are upscaled (*I*_*m*_ > 1). These targets are typically approached over relatively long periods of time (hours to days), and there is evidence that the feedback triggering these changes occurs largely during sleep [60,61].

Why might neurons use an MIMD rule to stabilize their activities? First, multiplicative weights ensure that the relative strengths of synapses are preserved, which is believed to help maintain specificity of the neuron’s response caused by learning. For example, if a neuron has three synapses with weights 1.0, 0.6 and 0.2, and if the neuron is firing above its target rate, then the new weights would be downscaled to 0.5, 0.3 and 0.1, assuming a multiplicative factor of *D*_*m*_ = 1/2. Thus the first synapse remains five times stronger than the third synapse, while pushing the firing rate of the neuron closer towards its target. While the sum of the *r*_*i*_’s immediately after synaptic scaling is applied is clearly different from their sum before scaling is applied, on average over long time scales and enough repetitions of this rule, neurons preserve a lognormal distribution of synaptic weights with approximately the same mean [58,59]. Thus, MIMD preserves efficiency, i.e. that the sum of all the weights *r*_*i*_ is approximately constant.

#### 3.3.3. Spike-timing-dependent plasticity (figure 3*c*)

STDP is a fundamental mechanism for associative learning in the brain [62]. The basic idea is the following: say, there are two connected neurons *u* → *v*. If neuron *u* fires and drives *v* to fire as a result, then a relationship between the two neurons is formed. This mechanism allows ‘concepts’ to be linked and associative memories to be formed.

In traditional Hebbian models, the relative timing of pre- and post-synaptic neurons (*u* and *v*, respectively) is used to modify the weight of the synapse, *r*_*i*_, between them. This change can be mediated by numerous molecular mechanisms, including changing pre-synaptic release probability or the number of post-synaptic receptors [63]. If firing of the pre-synaptic neuron immediately precedes the firing of the post-synaptic neuron, then feedback from post to pre (e.g. via a backpropagating action potential) results in an increase in synapse weight, called long-term potentiation (LTP). On the other hand, if the post-synaptic neuron fires after the pre-synaptic neuron, then the synapse undergoes long-term depression (LTD), decreasing its weight. The classic experiment by Bi & Poo [62] shows how the change in synaptic weight is a function of the delay in feedback from the time *u* fires to when *v* fires. Commonly, the maximum negative weight change is observed when the post-synaptic neuron fires immediately before the pre-neuron; however, since neurons repeatedly fire, this is equivalent to the longest delay possible between pre- and post- firing.

The effect of this time-dependent feedback is to minimize latency (i.e. the delay between when *u* fires and *v* fires) and drive the delay of a signal through the synapse to the minimum possible time. Synapses with the lowest latency experience LTP, and the increased weight, under the leaky integrate-and-fire neuron model [64]—where incoming synapses add weight to a leaky bucket, which causes the post-synaptic neuron to fire when full—allows the firing of *u* to contribute more to the firing of *v*. This results in additional LTP, pulling the firing of *v* closer to the firing of *u*. Overall, this process drives control of output neural firing to these low-latency neurons.

Though controversial, many models of Hebbian learning use a total weight limit, $\sum_{i}r_{i}=C$ [65]. Naturally, this is a competitive system whereby the lowest latency synapses gain weight at the expense of those with longer delays.

But how much do synaptic weights increase or decrease following feedback? There are two components that determine the new weight of a synapse. The first is the latency-sensitive component. Experimental evidence by Bi & Poo [62] and Zhou *et al.* [66] indicates that the latency-dependent change is MIMD, as would be expected if there is competition among neurons [65]. The second component is based on the current synaptic weight. For this, van Rossum *et al.* [67] propose adding an AIMD term, where smaller synapses experience a larger relative potentiation than larger synapses [68], whereas the relative depression is the same for all synapse sizes. In addition, this model eliminates competition among synapses by removing the constraint on the total synaptic weight limit (*C*); re-introducing competition requires an additional mechanism, such as synaptic scaling.

Further work is needed to explore how these three models—latency-sensitive MIMD, weight-sensitive AIMD and MIMD-based synaptic scaling—operate together and what network goals they attempt to satisfy. A tantalizing connection of biology and engineering can be found in the exponential weight-modification function measured in latency-sensitive STDP [65]. The characteristics of the exponential suggest a quasi-linear change for small latency, transitioning to a multiplicative increase at high latency. A popular variant of TCP, called CUBIC [69], uses a similar curve to blend AI and MI characteristics on the Internet, which we explore further in Discussion.

## 4. Discrete-event feedback in engineering

Below, we describe two examples of how discrete-event feedback control is instantiated in engineering. The first example, the regulation of data flow on the Internet, demonstrates how AIMD is used in a distributed system where efficiency and fairness are desired. The second example, weight update in machine learning, shows how MIMD is optimal when efficiency is desired but competition occurs among agents.

### 4.1. The transport control protocol on the Internet (figure 4)

**Figure 4.**
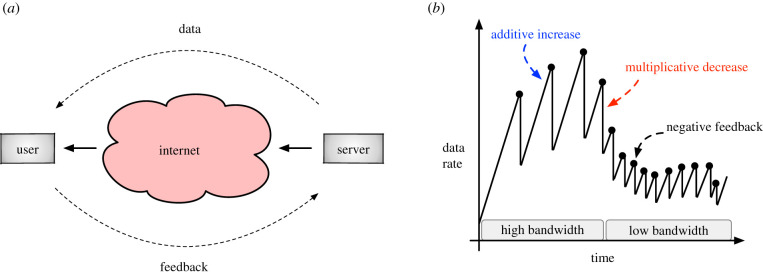
Transmission control protocol (TCP). (*a*) A server sends data to a user through the Internet. The user provides feedback acknowledgement to the server upon the receipt of each packet of data. When Internet congestion occurs, packets are lost or significantly delayed, causing the user to transmit negative feedback to the server. (*b*) Example data transmission rate of the server over time using the additive-increase multiplicative-decrease (AIMD) model. When packets are successfully transmitted, the server additively increases transmission rate (blue text). When packets are lost, the transmission rate is multiplicatively decreased (red text) in response to negative feedback (black dots).

The most familiar engineering application of discrete-event feedback regulation is the transport control protocol (TCP), used for congestion control on the Internet [70–72]. The Internet consists of billions of agents who send discrete packets of data (e.g. as part of a file) to each other over shared networking links (figure 4*a*). Agents desire to transmit their data as quickly as possible; however, each individual link can only handle limited traffic at a time. In this example, there are *n* agents on the Internet, and each agent *i* is sending data to another user at a rate of *r*_*i*_. *C* corresponds to the total bandwidth available that all users must share.

With a network as broad as the Internet, a given link is only needed by a minuscule fraction of agents at any time, so models are simplified to the sharing of a single bottleneck link. Packets travel from one agent to another through routers, connected to each other via links with limited bandwidth. If packets are destined for a link that is already fully used, packets will first be queued into a buffer in the router. If the demand for the link persists, the buffer will fill to its maximum capacity and subsequent packets will be discarded. This link is then said to be congested.

The goal of the system is to achieve efficiency (i.e. $\sum_{i}r_{i}=C$ so that bandwidth is neither over- nor under-used) and to achieve fairness (i.e. all the *r*_*i*_’s are equal, or approximately equal), so that all agents are treated the same. Of course, no agent is privy to the values of *C* and *n*, both of which vary with time.

How is TCP feedback control used to solve this problem? If a packet is not lost due to congestion, it will reach the destination agent, which will then respond with a 1-bit positive feedback signal, called an acknowledgement packet (ACK). When the origin agent receives the ACK, it infers that *C* has not yet been collectively reached and will thus additively increase the rate of transmission, often by one additional packet per interval. If, on the other hand, a packet encounters congestion and is discarded, the destination agent will detect a missing packet from the transmission sequence, and it will send a negative feedback signal, called a negative acknowledgement (NAK). A NAK implies that the shared networking links are over-burdened, and as a result, the origin agent applies a multiplicative decrease, usually by cutting the rate in half. The algorithm is simply

$$r_{i}^{t+1}=\left\{ \begin{array}{cc} r_{i}^{t}+I_{a}\; & \text{if an ACK is received}\;\\ r_{i}^{t}\times D_{m}\; & \text{if a NAK is received},\; \end{array}\right.$$

where typical settings are *I*_*a*_ = 1, *D*_*m*_ = 1/2 [7,73,74].

TCP regulates the *r*_*i*_ based solely on communication between the origin and destination agents and does not require explicit processing by routers along a link. Thus, this AIMD algorithm is highly scalable and adaptive, as transmission rates naturally adjust as agents join or leave the system. Later, we describe additional variants of this basic algorithm, and other optimization goals that are aligned with those observed biologically.

### 4.2. Multiplicative weight updates in machine learning

One of the most common and simplest algorithmic techniques for decision-making in machine learning is called the multiplicative weights update method [75]. Imagine making a binary decision each day (e.g. whether to buy a stock) based on the day-to-day opinions of *n* experts. It is unknown *a priori* which experts are the most reliable, and this information is only revealed over time. Specifically, each expert *i* is assigned an initial weight $r_{i}^{t}$, indicating the confidence in the expert’s opinion on day *t*. The distribution of the $r_{i}^{t}$ is defined such that $\sum_{i}r_{i}^{t}=C=1\;\forall t$; i.e. some fraction of the total confidence is allocated to each expert. At the end of each day, positive feedback is provided for correct experts, and negative feedback is provided for incorrect experts, based on their opinion on that day. If expert *i* was correct, then the value of *r*_*i*_ should be increased, and vice versa if expert *i* was incorrect. The goal is to converge over time to the setting of weights (*r*_*i*_’s) that maximize profit when following the experts’ advice.

The multiplicative update algorithm is simple:

$$r_{i}^{t+1}=\left\{ \begin{array}{cc} r_{i}^{t}(1+\epsilon )\; & \text{if expert }i\text{ is correct on day }t\;\\ r_{i}^{t}(1-\epsilon )\; & \text{if expert }i\text{ is incorrect on day }t,\; \end{array}\right.$$

where *ε* is a small positive constant (e.g. *ε* = 0.01). After each day, all the *r*_*i*_ are re-normalized to ensure they sum to 1. Thus, in this problem, confidence is always completely allocated over the experts, i.e. efficiency is guaranteed.

Why is MIMD used to update weights? One property is that MIMD separately preserves the relative ratio of confidences within each group of correct and incorrect experts on each day [75] and therefore retains knowledge from previous days. Strikingly, the multiplicative iterative update rule converges, within bounded error, to the optimal set of weights [76].

Perhaps due to its simplicity, the multiplicative weight update method has been re-discovered numerous times across fields. For example, in machine learning, multiplicative weights serve as the basis for the popular adaptive boosting algorithm [77], which combines multiple weak experts into a single output prediction.

## 5. Goals of AIMD and MIMD systems

In the previous sections, we saw how AIMD and MIMD are used in broad biological and engineered systems. In this section, we delve deeper into the specific goals that are optimized by different feedback control algorithms (table 1) to help explain why these algorithms are so prevalent compared to alternatives.

### 5.1. Efficiency

Optimizing efficiency is a primary goal of all feedback control systems we have discussed. For example, how much excess data is sent that is never received? How many ants forage and return home empty-handed? Formally, we call a system optimally *efficient* when

5.1 $$\sum_{i=1}^{n}r_{i}=C.$$

If $\sum_{i}r_{i}>C$, then more data are sent than can be delivered, or more ants are deployed compared to food available—the system is overloaded. On the other hand, if $\sum_{i}r_{i}<C$, performance can be further improved without downside: otherwise, for example, neurons would be hypoactive, providing little useful information, and cell growth would be slow, wasting available nutrients. Both cases are undesirable.

While simple, this goal is challenging to meet in decentralized systems because each user *i* is only privy to its own control variable *r*_*i*_, yet the goal is for the collective settings of all the *r*_*i*_ to satisfy equation (5.1). Furthermore, *C* is not explicitly known and can change over time. This applies to all of our example systems, except multiplicative weights update, which is a centralized system.

**Figure 5.**
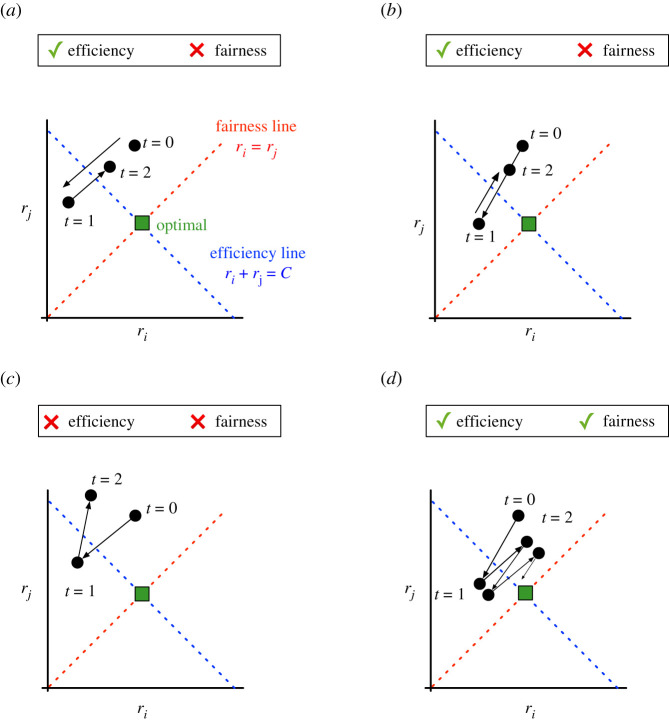
Illustration of how four algorithms converge to efficiency and fairness. Each panel shows the evolution of *r*_*i*_ (*x*-axis) and *r*_*j*_ (*y*-axis). The dotted red line corresponds to perfect fairness, where *r*_*i*_ = *r*_*j*_. The dotted blue line corresponds to perfect efficiency, where *r*_*i*_ + *r*_*j*_ = *C*. The green square is the point where both fairness and efficiency are both optimized. See text for comparison of each algorithm: (*a*) AIAD, (*b*) MIMD, (*c*) MIAD and (*d*) AIMD.

In all four algorithms (AIMD, AIAD, MIMD, MIAD), each user *i* constantly increases or decreases *r*_*i*_ on receipt of feedback. Figure 5 illustrates how well each algorithm achieves efficiency using a vector notation originally presented by Chiu & Jain [7]. For simplicity of analysis, the system contains only two users *i* and *j*, so the two axes show the values of *r*_*i*_ and *r*_*j*_. We also assume that both users receive the same feedback, and thus, apply the same increase or decrease rule in each time point, simultaneously. Each panel shows the evolution of the rates *r*_*i*_ and *r*_*j*_, starting from the initial operating point $\left(r_{i}^{0},r_{j}^{0}\right)$ at time *t* = 0. The blue line shows where perfect efficiency is achieved, corresponding to the line *r*_*i*_ + *r*_*j*_ = *C* with a slope of −1.

AIAD (figure 5*a*) shows the effect of additive changes: the operating point moves along a vector with slope 1, perpendicular to the efficiency line, since the same factors (*I*_*a*_ and *I*_*d*_) are being added or subtracted from each rate simultaneously. While AIAD will converge around a point on the efficiency line, it does so in a slower, linear fashion. For MIMD (figure 5*b*), since the changes are multiplicative, the operating point remains along a line formed by the origin and initial operating condition, and converges to efficiency exponentially in time.

For MIAD (figure 5*c*), the decrease vector is perpendicular to the efficiency line, whereas the increase vector follows the line through the origin, and vice versa for AIMD (figure 5*d*). Thus, for MIAD, the operating point converges to the upper left or bottom right corners. This rule essentially implements a ‘winner-take-all’, where a single user consumes all the resources. Uniquely, AIMD is the only algorithm that converges exponentially towards the centre of the plot, where it approaches the efficiency line.

A further analysis of the four rules [78] showed analytically how much a system ‘overshoots’ *C* when a second user is added to a steady-state system. For typical increase and decrease constants (*I*_*a*_ and *I*_*d*_), AIMD has the least overshoot followed by typically AIAD, MIMD, then MIAD. A centralized control system, where *C* is known, can easily achieve efficiency with no convergence time or overshoot, as in the case with the normalization step in the multiplicative weights update example.

### 5.2. Fairness

For systems with users that cooperate (not compete) for resources, the second property desired is for each user to acquire an equal share of the resource *C*. For example, on the Internet, no user should be prioritized, and all users should obtain approximately the same bandwidth. A system is optimally cooperative or *fair* when:

5.2 $$r_{1}=r_{2}=\cdots =r_{n}.$$

In figure 5, the red line shows where perfect fairness is achieved, corresponding to the line *r*_*i*_ = *r*_*j*_ with slope 1. From the evolution of the vectors, it is evident that AIAD and MIAD do not converge to the fairness line. For AIAD (figure 5*a*), the operating point can only move along a vector parallel to the fairness line. MIAD (figure 5*c*) is worse: it only travels away from the fairness line, such that the user with the initially higher rate will eventually gain all of the resource; i.e. it converges to unfairness. MIMD (figure 5*b*) moves along a vector through the origin, and thus only reaches the fairness line in the trivial case where both *r*_*i*_ and *r*_*j*_ are zero. However, MIMD preserves a notion of ‘relative’ fairness useful is competitive scenarios, which we discuss in the next section.

Strikingly, AIMD (figure 5*d*) is the only algorithm that converges to fairness since it alternates between the AI directions, perpendicular to the fairness line, and MD trajectories, which pass through the current operating point and the origin. Coupled with the results above, AIMD is the only algorithm that converges near a single point where efficiency and fairness are both optimized [73]. In fact, the point to which AIMD converges is globally unique and exponentially stable [74]. While this stability can be compromised by excessive feedback delays [72], this remarkable algorithm from 1988 [71] was quickly adopted for computer networking applications, and it remains at the core of the congestion control algorithm used on the Internet today. For such a global-scale network, there are many variants of TCP in use, some of which are discussed later, as well as non-AIMD protocols. These can attempt to gain more bandwidth (e.g. prioritizing voice conversations) or deprioritize themselves (downloading large software updates), so strict fairness or efficiency is not guaranteed. Nonetheless, as new algorithms are developed, the property of *TCP-friendliness* is actively sought, meaning that the fairness and efficiency of existing traffic are not significantly harmed when shared links become congested.

We thus find it quite elegant that this strategy has arisen widely in biology via evolution for diverse problems, such as cellular size homeostasis, to ensure cell sizes are uniform, and for novelty detection in the brain, to ensure novel odours are all equally detected.

### 5.3. Competition

In competitive scenarios, users do not wish to share resources equally (fairly), but rather to gather as many resources (*C*) for themselves as possible. However, this is a zero-sum game: for one user to gain more share, another user must lose share. If users always retain their proportional share and do not cede any, then the relative distribution of resources cannot change: we denote this as preserving the *degree of fairness*. Formally, Chiu & Jain [7] defined a degree of fairness index *F* as

5.3 $$F=\frac{{\left(r_{i}+r_{j}\right)}^{2}}{2(r_{i}^{2}+r_{j}^{2})},$$

where *F* = 1 is perfectly fair and *F* = 1/2 is perfectly unfair, when one of the two users acquires all of the resources. For *n* users, *F* can be extended to $F={\left(\sum_{i=1}^{n}r_{i}\right)}^{2}/(n\sum_{i=1}^{n}r_{i}^{2})$, and *F* = 1/*n* denotes perfect unfairness.

How do additive and multiplicative rules affect the degree of fairness? If *r*_*i*_ and *r*_*j*_ are both multiplied by the same constant (i.e. MI or MD), then *F* is unchanged. In figure 5*b*, this corresponds to lines that travel through the origin, called *equi-fairness* lines. However, adding a constant to both *r*_*i*_ and *r*_*j*_ (i.e. AI or AD) changes *F*: positive constants (AI) increase relative fairness, while negative constants (AD) decrease it.

For harvester ants, a colony is not advantaged by ceding resources to other colonies, so the fair nature of AIMD is not desired. MIMD is preferred because it both preserves the degree of fairness at all times and optimizes efficiency. Like AIMD, MIMD is stable. This assumes that all colonies receive simultaneous and identical feedback, which is of course not always true in practice; e.g. one colony might find food while another colony may not, and thus one colony receives positive feedback while the other receives negative feedback. This is a means whereby the degree of fairness changes, among other potential non-ideal conditions [6].

The situation is similar for the multiplicative weights update problem. Here, MI is applied to experts that predict correctly, while applying MD to those in error. The relative fairness within each group of correct or incorrect experts is preserved by the multiplicative update, while the overall fairness is adjusted to promote correct experts. The subsequent vector normalization, where each weight is divided by the sum of the weights such that the vector sums to 1 (which guarantees efficiency) applies a constant multiplicative factor to all components, and therefore does not change the degree of fairness from the previous step. Finally, for homeostatic weight plasticity in the brain, MIMD preserves the relative ratios of synapse strengths; this is believed to be important for maintaining learning specificity, while adjusting the operating point of the neuron to a more efficient position.

Why is not MIAD used instead of MIMD? MIAD converges to perfect unfairness by allowing the user with the greatest initial share to eventually consume 100% of the resources. Any user without the initial maximum share would not choose this algorithm since it would result in loss of all of its resources. To counter, they may utilize a grossly inefficient algorithm to wrestle share, such as maximizing *r*_*i*_ by sending all ants out at once (known as bang-bang control). MIMD instead avoids any loss of proportional share, while preserving efficiency for all. Thus, evolutionarily, despite competition, some latent cooperation may be required to preserve the existence of a population [79,80].

Finally, competitive systems usually have a temporal aspect that necessitates aggressive responses to feedback. For ant foraging, seeds should be collected as soon as possible to reduce environmental loss, making the aggressive rise of MI desirable, particularly over AI. The MD term is also necessary to conserve resources by aggressively scaling back foraging when supplies become scarce, or more importantly, when foragers are under attack. We previously showed [78] that MIMD can reach full utilization of resources faster than AIMD, at the expense of temporarily overshooting *C*.

### 5.4. Latency

In the three engineering and biological applications that we detailed—harvester ants, TCP on the Internet, and STDP in the brain—feedback delay (called latency) is used as information for optimization, rather than a property of the feedback algorithm itself. For harvester ants, latency corresponds to the time each ant spends foraging for food; as this time increases, ants queuing in the nest can infer that seeds are becoming scarce and can thus pre-emptively reduce foraging rates to conserve water.

Strikingly, a similar algorithm was independently developed for congestion control on the Internet. By measuring the time between the transmission of a packet and the receipt of its ACK, significant increases in latency can be detected, suggesting that packets are waiting in queues for fully used links. This serves as a warning of incipient congestion in modern TCP variants, including TCP Vegas [81] and Compound TCP [82]. By reducing transmission rate before congestion causes packet loss, these protocols conserve network resources which would otherwise be wasted when a packet is dropped midway through transit.

In the brain, STDP aims to minimize feedback delays by strengthening the weight of synapses between neurons that fire coincidentally. This effectively serves two purposes. First, it helps reduce reaction time between when sensory signals are received and when behaviours are triggered in response (useful, for example, when hitting a baseball). Second, it helps to form strong associative memories, whereby neurons that are co-active are linked functionally.

In this context, algorithms that converge to efficiency will also minimize latency: for ants and TCP, this is accomplished by avoiding overload; in the brain, latency is measured and minimized by the feedback algorithm. Therefore, both AIMD and MIMD will act to minimize latency, under the same performance and overshoot findings discussed earlier.

### 5.5. Slow start

How do systems efficiently begin activity from a quiescent state, for example, when harvester ants begin foraging in the morning, when a new connection is established over the Internet, or when cells first begin to grow in size? In most cases, the initial *r*_*i*_ are a small fraction of the values which they will eventually reach, so the *r*_*i*_ can aggressively increase to speed convergence, improving overall efficiency.

Both the Internet and cell sizing mechanisms have adopted the same solution: initially applying multiplicative increase instead of additive increase. In TCP, this is called *slow start*, and it operates until a preset transmission rate is reached or when congestion is detected [70,72,81]. Similarly, during the initial cell cycle in plants, cell sizes first increase multiplicatively, before transitioning to additive growth later in the cell cycle [33].

Slow start is also applied after no feedback is received for an extended period of time. For ants, *timeout* behaviour occurs when the departure of foragers ceases after no forager returns for 20 min (figure 1*b*) [18]. This behaviour is believed to be a protection against predators, which stand along trails consuming foragers. Eventually, a small number of patroller ants leave the nest and must successfully return to the nest before MIMD-based foraging restarts. Similarly, on the Internet, if a packet is not acknowledged within five times its estimated latency, then severe congestion or link failure is assumed by TCP, *r*_*i*_ is reset to its initial condition, and slow start begins again.

## 6. Discussion

### 6.1. Summary

Biological and engineered systems exploit discrete-event feedback as a robust, scalable and lightweight means of regulating activity. This feedback often occurs as discrete events: a harvester ant returning to the nest or an acknowledgement of successful data transfer. The basic rules used to adjust activity in response to feedback can be additive (adding or subtracting a constant upon receipt of positive and negative feedback, respectively), or multiplicative (multiplying or dividing a constant). We showed that out of four possible combinations of these algorithms, AIMD and MIMD are found in biological and engineered systems. Both of these algorithms lead to efficient behaviour, where resources are neither over- nor under-used. The two algorithms differ in that AIMD converges to fairness, where each user acquires an equal share of the available resources, whereas MIMD does not modify the existing level of fairness. AIMD is thus an attractive strategy in cooperative systems, whereas MIMD is typically found in competitive systems. In addition, we described advanced techniques that some systems use to adapt their behaviour, including using feedback delay (latency) to anticipate future problems, and using slow start to quickly ramp up activity upon initialization or when recovering from failures (timeout).

### 6.2. Complexities of biological systems and opportunities for theorists

Biological systems also introduce new twists on traditional feedback control problems that may motivate improved algorithm design. We highlight a few such twists below:

1. *Noise*. Feedback can be noisy due to errors, perturbations, or even adversaries, and it is not clear how AIMD and MIMD act to reduce effects of these errors. In engineering, discrete, digital systems tend to have superior performance under low noise levels, but degrade rapidly at high noise levels, compared to continuous analogue systems. Relatedly, AIMD only guarantees fairness when all nodes play by the same rules. How do biological systems account for noise, and how do they detect unfair competitors (e.g. mutants) in otherwise cooperative scenarios? These issues are known to have adverse effects; e.g. perturbing cell size homeostasis mechanisms can lead to cancer. Analogous challenges remain outstanding in engineering, especially in cybersecurity.
2. *Adaptation in parameters.* Feedback parameters (i.e. *I*_*a*_, *I*_*m*_, *D*_*a*_, *D*_*m*_) could adapt over time or be context-dependent. Indeed, more complex feedback rules are used in engineering, such as replacing the fixed increase–decrease constants with arbitrary functions that may depend on the present rate or past activity. For example, on the Internet, TCP CUBIC replaces fixed AIMD constants by initially increasing *r*_*i*_ using additive-increase and then transitioning to multiplicative-increase via an aggressive time-dependent cubic function [69].
  In biology, we observed a similar hybrid strategy in cell sizing mechanisms, and in the brain, Oja [83] proposed a functional weight update rule for Hebbian learning, where the synaptic weight increase depends on the current weight, instead of utilizing a fixed constant. In addition, global modulators, such as sleep–wake signals or danger–stress signals, may modify behaviour to temporarily prioritize certain goals. Along with robustly adapting feedback, transitioning between states while avoiding sudden jumps and oscillation is a challenge in engineering. Biological systems may yield clues on how to implement this without the complex, centralized logic currently needed in engineered systems.
3. *Spatial constraints.* We assumed that each user receives and applies identical, simultaneous feedback, and that there is no feedback delay. In practice, these approximations may not hold true, particularly in large, distributed systems. TCP CUBIC is one such algorithm developed from active research in *long fat networks*, which are Internet connections with very high feedback latency. In biology, nodes are distributed in space, lack unique identifiers, and often lack mechanisms to provide precise, node-to-node feedback [84]. These constraints introduce new challenges for optimization that are reminiscent of those faced in stone-age computing protocols [85] and gossip protocols (also known as epidemic protocols) [86,87], where information is passed between users in a decentralized manner. Understanding how to deal with outdated, missing or erroneous feedback under these protocols is an area of active research.

### 6.3. Future work and guidance

While many well-studied examples of feedback control in biology appear to use continuous analogue control, as opposed to discrete feedback [1], other directions to explore include synthetic biology [88] and transcriptional regulation and molecular switching [4]. Biological systems also combine continuous and discrete variables, known in engineering as hybrid dynamical systems [89], as found in gene regulation, bacterial chemotaxis and sleep–wake regulation [90].

How might this framework be used to study other problems of interest? First, two variables need to be defined: a constrained variable *r*_*i*_ for each agent, and a total amount of capacity or resource, *C*. At the core of the problem is the regulation of *r*_*i*_ such that $\sum_{i}r\leq C$. Second, the discrete feedback needs to be identified—when is it triggered, what triggers it, how is it transmitted—which is used to adjust the *r*_*i*_ variables over time. Third, the goals need to be defined, including whether the agents seek efficiency, and if they are cooperating (fairness) or competing. Finally, the algorithm needs to be determined, including how the *r*_*i*_ variables respond to feedback—for example, via additive, multiplicative, functional, time-, history- and latency-dependent updates—and whether other features are used, such as slow start and timeout. If goals are unknown, determining the response to feedback can suggest putative goals, and vice versa: if the algorithm is unknown, the goals may suggest likely response.

## Data Availability

This article has no additional data.
